# Perception of risk of exposure in the management of hazardous drugs in home hospitalization and hospital units

**DOI:** 10.1371/journal.pone.0253909

**Published:** 2021-07-01

**Authors:** Mari Ángeles Bernabeu-Martínez, Julia Sánchez-Tormo, Pedro García-Salom, Javier Sanz-Valero, Carmina Wanden-Berghe

**Affiliations:** 1 Department of Public Health and History of Science, School of Medicine, Miguel Hernandez University, Elche, Spain; 2 Pharmacy Service, University General Hospital, Sant Joan d’Alacant, Spain; 3 Faculty of Health Sciences, Universitat Oberta de Catalunya, Barcelona, Spain; 4 Pharmacy Service, University General Hospital, Alicante, Spain; 5 National School of Occupational Medicine, Carlos III Health Institute, Madrid, Spain; 6 Health and Biomedical Research Institute of Alicante, University General Hospital, Alicante, Spain; Duke University, UNITED STATES

## Abstract

**Objective:**

To assess the perception of risk of exposure in the management of hazardous drugs (HDs) through home hospitalization and hospital units.

**Material and methods:**

A questionnaire was released, at the national level, to health professionals with HD management expertise. Questionnaire included 21 questions that were scored using a Likert scale: 0 (null probability) to 4 (very high probability). The internal consistency and reliability of the questionnaire were calculated using Cronbach’s alpha and the intraclass correlation coefficient, respectively.

**Results:**

144 questionnaires (response rate 70.2%) were obtained: 65 (45.1%) were nurses, 42 (28.9%) occupational physicians, and 37 (26.1%) were pharmacists. Cronbach’s alpha was 0.93, and intraclass correlation coefficient was 0.94 (95% CI 0.91–0.97; p-value < 0.001). The mean probability was 1.95 ± 1.02 (median 1.9; minimum: 0.05; 1st quartile 1.1; 3rd quartile 2.6; and maximum 4). Differences were observed in scoring among professional groups (occupational physicians *versus* nurses (1.6/2.1, p = 0.044); pharmacists *versus* nurses (1.7/2.1, p = 0.05); and occupational physicians *versus* pharmacists (1.6/1.7, p = 0.785), due mainly to the administration stage (p = 0.015).

**Conclusions:**

The perception of risk of exposure was moderate, being higher for nurses. It would be advisable to integrate HDs into a standardized management system (risk management model applicable to any healthcare center) to improve the safety of health professionals.

## Introduction

Concern about the safe handling of drugs by health workers has been present since Falck et *al*. [1] detected, in 1979, mutagenicity in the urine of nurses who prepared cytostatics. This study was a milestone because for the first time, the existence of a health risk in cases of continuous exposure to some drugs was demonstrated, and from that moment, the concept of occupational exposure emerged.

The American Society of Hospital Pharmacists (ASHP) [2] introduced, in 1990, the term “hazardous drug” (HD), which until then was exclusively associated with cytostatics. Later, in 2004, *the National Institute for Occupational Safety and Health* (NIOSH) [3] adopted this terminology, giving rise to the internationally accepted definition today, which goes beyond the field of cytostatics: “any drug that presents, in humans, one or more of the following hazard criteria: carcinogenicity, teratogenicity or other developmental toxicity, reproductive toxicity, low-dose organ toxicity, or genotoxicity or drugs with structure or toxicity profiles similar to other hazardous drugs”.

HDs have been described as the greatest chemical hazard present in the health field and one of the most dangerous chemical agents ever developed [4]. Therefore, there are priority recommendations or strategies to improve safety that are developed by experts in workplace safety entities, such as the *Joint Commission* [5], Occupational Safety and Health Administration *(OSHA)* [6], the *Pan American Health Organization* (PAHO) [7] and the *European Agency for Safety and Health at Work (EU-OSHA)* [8].

It is evident, then, that the process of handling HDs, constituted by circuits of high complexity and with a large number of actors involved, involves important risks for health professionals, making it essential to ensure the safety of the process. Consequently, risk assessment becomes one of the key points in the management and control of the HD process.

In this regard, it is important to note that the HD handling guide published in 2007 by the *International Society of Oncology Pharmacy Practitioners* (ISOPP) [9] dedicates a specific section to risk assessment. In the US, *The Hazardous Drug Consensus Group* (HDCG) provides risk assessment as one of the key points in improving the safety of handling HDs because, as explained, the results will depend on preventive measures that the organization will adopt [10]. Finally, in Spain, the consensus document endorsed by different scientific societies, unions, groups of health managers, and patient associations urges the Ministry of Health to review and analyze critical points at different stages of the HD handling process [11].

Although the different stages of the HD handling process are carried out fundamentally within health centers through the services of pharmacies and different clinical units involved in patient care, it should be emphasized that exposure to risks occurs both during the stay in a health center and in homes (where exposure affects both professionals and family members) [12]. This is the case even more so when a patient is assigned to a hospitalization unit that administers the medication in the patient’s home and in which the conditions in the workplace (the patient’s home) are usually uncontrolled and lack, in most cases, direct supervision [8].

In this context, the recent work published by Bernabeu et al. [13] identified the hazards and described the chemical risks associated with the HD handling process, establishing the basis for future risk assessments.

Consequently, the objective of this study was to evaluate perception of risk of exposure in the management of HDs through HHUs and hospitalizations units.

## Materials and methods

### Study design

An electronic questionnaire was designed in Google Forms, with Likert scale response options, for distribution through the Internet.

The specific questionnaire was agreed upon by a group of experts consisting of four hospital pharmacists, one HHU physician, and one occupational health auditor pharmacist, taking previous work from Bernabeu *et al*. [14, 15] as a basis for the selection of the stages and dangers to be addressed.

The final questionnaire (S1 and S2 Tables) included an introduction (profession of the researched population: occupational physicians, nurse or pharmacist), 21 specific questions on the subject under study and a closing section and online submission of the document once completed.

The specific questions were classified into 9 sections: a) preservation (storage of the drug under proper conditions of temperature, light and humidity); b) waste management; c) transport to the place of administration; d) administration: previous stages; e) administration by intravenous (IV) infusion; f) subcutaneous (SC), intramuscular (IM), intrathecal (IT) and IV bolus administration; g) intravesical (IVe) administration; h) ophthalmic (OP) administration; and i) administration: final stages.

All items were scored using a Likert scale with scores from 0 to 4 (0 = null perception that exposure will not occur in any case; and 4 = very high probability: perception that exposure would always occur). In any case, it was indicated that those items for which the appropriate response was not known (due to lack of knowledge or experience in the specific operation or stage) were left unanswered.

As a final phase of the questionnaire design, a pilot-pre-test was performed by allotting the questionnaire in paper format to a group of 12 professional experts in HDs, belonging to the different authors workplaces in order to ensure that the wording of the questions could be understood by the respondents, making the correction considered appropriate. Finally, a test form was completed to confirm that data was correctly recorded in the Excel file linked to the Google form.

Cronbach’s α coefficient and the intraclass correlation coefficient (ICC) were calculated as measures of internal consistency and reliability of the questionnaire (validation of the questionnaire).

### Population and scope of the questionnaire

The target population was health professionals (occupational physicians, nurses and pharmacists) with recognized experience in the management of HDs, either for their care work (both at the hospital, outpatient or home level) or for their technical knowledge in the field. We decided to survey the main profiles of health professionals involved in the managing of HDs, who are responsible for carrying out the main international guidelines for handling HDs [14], in order to procure results that reflect an integrated multidisciplinary vision (both technical-theoretical and technical-practical knowledge). Additionally, this allows us to evaluate differences in risk perception between them, with the ultimate aim of better understanding real risk and carrying out a more accurate risk assessment in subsequent works.

The size of this population was calculated based on the main objective of this study and the estimation of Cronbach’s α coefficient and the intraclass correlation coefficient (ICC) as measures of internal consistency and reliability of the questionnaire. To calculate the minimum necessary number of respondents, the expected values of both coefficients were assumed to be those obtained in previous experiences (0.90 for both statistics), a confidence level of 95% and a precision or amplitude of the interval of 5%. It was necessary to include a minimum of 142 people [16]. These calculations were determined from Bonett’s formulas [17, 18].

### Implementation of the questionnaire

The questionnaire was disseminated nationally via email, which included a link of the electronic questionnaire, to a list of recruited professionals belonging to the Spanish group for the development of oncology pharmacy (Grupo Español para el Desarrollo de la Farmacia Oncológica—GEDEFO) of the Spanish Society of Hospital Pharmacy (Sociedad Española de Farmacia Hospitalaria—SEFH), to the management and quality working group of the Spanish Society of Home Hospitalization (Sociedad Española de Hospitalización a Domicilio—SEHAD) and to the professionals assigned to occupational risk prevention and preventive medicine services through the National School of Occupational Medicine (Escuela Nacional de Medicina del Trabajo—ENMT).

The questionnaire was conducted from November 18, 2019, to February 18, 2020, sending a reminder every month through the same distribution list.

### Information processing

The data were collected in an Excel table in Google Forms, periodically making backup tables to safeguard the information. In cases where deviations and inconsistencies were detected, corrections were made by consulting the original table.

### Perception of risk of exposure

To determine the perceived risk of exposure, the following ranges were determined: low risk from 0 to 1; moderate risk from 1.1 to 2; high risk from 2.1 to 3; and very high risk from 3.1 to 4.

### Distribution of the results

The respondents were able to edit their answers after submitting if they detected an error or wanted to modify any of their answers. Likewise, after the final submission, they could see summary graphs for the set of results and for each item.

### Statistical analysis

For the qualitative variable (profession), the absolute and relative frequencies (percentage) were calculated. For the quantitative variables (the 21 items in the questionnaire), the mean and standard deviation, the median, the maximum and minimum, and the first and third quartiles were obtained.

To analyze the properties of the questionnaire and the relationships between its elements, the internal consistency was checked using Cronbach’s alpha.

To quantify the reliability of the measurements associated with the continuous quantitative variables (items), the ICC and its 95% confidence interval were calculated (using the two-way random-effects model and the multiple raters’ type).

To verify the normality of the variables, the Kolmogorov-Smirnov test (with Lilliefors correction) was used. The comparison of the medians between groups was performed using the Kruskal-Wallis test, studying the association between groups with the Wilcox test and the Benjamini & Hochberg method.

The level of significance used in all hypothesis tests was α ≤ 0.05.

For the data analyses, R version 3.6.3 for Windows was used.

The datasets generated during and/or analysed during the current study are available from the corresponding author by reasonable request.

### Ethical considerations

The questionnaire and methodology for this study were approved by the Ethics Committee of the University General Hospital (Ethics approval number: CEIC PI2017/93). Informed consent was not required to participate in the questionnaire from the participants since it was completely an anonymous and volunteer questionnaire and no personal data were obtained. The professionals were recruited through distribution lists of the participating groups/societies. Administrator of those societies gave their consent prior to the distribution of the link to the questionnaire, given the scientific interest of this work.

## Results

Responses from a total of 144 professionals who met the criterion of having experience in the management of HDs were obtained: 65 (45.1%) nurses, 42 (28.9%) physicians (occupational physicians) and 37 (26.1%) pharmacists, representing a response rate of 70.2%.

Of the returned questionnaires, 115 (79.9%) were complete; items Q13, with 12 missing values, and Q11 and Q8, each with 9 missing values, accounted for the items with the most missing values.

Regarding the consistency of the questionnaire, the Cronbach’s alpha was greater than 0.9 in all cases. In addition, regarding the homogeneity of each item individually, a value greater than 0.9 was obtained for all items. Consequently, an analysis of the disturbances showed that it was not necessary to eliminate any of the items. The reliability of the questionnaire, measured by the ICC, was 0.94 (95% CI 0.91–0.97 and p-value < 0.001; see Table 1).

**Table 1:** Measures of internal consistency and reliability.

	Internal Consistency	Reliability
	α Cronbach	Correlation coefficient Intraclass (95% CI)
Questionnaire	0.93	0.94 (0.91–0.97)
Questionnaire by occupational physicians	0.94	0.80 (0.70–0.89)
Questionnaire by nurses	0.93	0.87 (0.79–0.93)
Questionnaire by pharmacists	0.91	0.86 (0.79–0.93)

The normality test (Kolmogorov-Smirnov test, with Lilliefors correction) indicated that the sample was not normally distributed, both in the overall results and in the stages; therefore, we used nonparametric population comparison tests (global 3.52e-03; preservation 6.25e-41; waste management 3.66e-10; transport 3.79e-12; and administration 4.97e-02).

### Results of the perception of risk of exposure to HDs

The analyses of the questionnaire responses resulted in the following central measures: mean 1.95 ± 1.02; median 1.9; minimum 0.05; 1st quartile 1.1; 3rd quartile 2.6; and maximum 4. The statistics for each item are provided in Table 2, as well as the statistics for different stages of the process and routes of administration.

**Table 2:** Central measures for the 21 items in the questionnaire and the statistics on perception of risk of exposure according to the different sections of the questionnaire.

Item	Minimum	1st quartile	Median	Mean ± SD	3rd quartile	Maximum	Perception of risk of exposure
Q1	0	1	1	1.6 ± 1.1	2	4	Middle
Q2	0	1	2	2.1 ± 1.2	3	4	High
Q3	0	1	1	1.2 ± 0.9	1	4	Middle
Q4	0	1	2	1.8 ± 1.1	2	4	Middle
Q5	0	1	2	1.6 ± 1.1	2	4	Middle
Q6	0	1	2	2.2 ± 1.1	3	4	High
Q7	0	2	2	2.4 ± 1.0	3	4	High
Q8	0	1	1	1.5 ± 1.0	2	4	High
Q9	0	1	2	1.9 ± 1.0	3	4	Middle
Q10	0	2	3	2.8 ± 1.0	4	4	High
Q11	0	1	2	1.8 ± 0.8	2	4	Middle
Q12	0	1	2	2.1 ± 1.1	3	4	High
Q13	0	1	2	1.9 ± 0.9	3	4	Middle
Q14	0	1	1	1.5 ± 0.9	2	4	Middle
Q15	0	1	2	2.3 ± 1.1	3	4	High
Q16	0	1	2	2.1 ± 1.0	3	4	High
Q17	0	1	2	2.3 ± 1.1	3	4	High
Q18	0	1	2	2.2 ± 1.1	3	4	High
Q19	0	1	2	2.0 ± 1.0	3	4	Middle
Q20	0	1	2	1.9 ± 1.1	3	4	Middle
Q21	0	1	2	1.9 ± 1.0	2	4	Middle
Preservation	0	1	1	1.5 ± 1.0	2	4	Middle
Waste manag.	0	1	2	1.9 ± 1.1	2.5	4	Middle
Transport	0	1	1	1.3 ± 1.0	2	4	Middle
Administration	0	1	2	2.0 ± 1.1	3	4	Middle
Administration IV	0	1	2	2.0 ± 1.1	3	4	Middle
Administration SI	0	1	2	2.1 ± 1.1	3	4	High
Administration IVe	0	1	2	2.2 ± 1.1	3	4	High
Administration OP	0	1	2	2.0 ± 1.1	3	4	Middle
Global	0.05	1.1	1.9	2.0 ± 1.0	2.6	4	Middle

Waste manag. = Waste management

IV = Intravenous perfusion; SI = Subcutaneous, intramuscular, intrathecal, intravenous bolus: IVe = Intravesical; OP = Ophthalmic.

**Fig 1:**
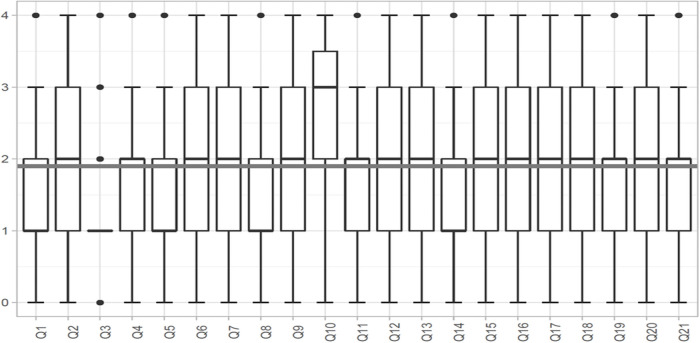
Graphical representation of the distribution of the responses for the 21 items in the questionnaire.

A graphical representation of the distribution of the responses to the 21 questions, using a boxplot, can be seen in (Fig 1).

### Perception of risk of exposure according to professional group

The comparison of the medians by the Kruskal-Wallis test among the 3 groups of professionals showed significant differences (p = 0.021). The Wilcox test, with the Benjamini & Hochberg method, resulted in the following associations: occupational physicians *versus* nurses (1.6/2.1, p = 0.044); pharmacists *versus* nurses (1.7/2.1, p = 0.05); and occupational physicians *versus* pharmacists (1.6/1.7, p = 0.785). If there were differences among the professionals, according to the different sections of the questionnaire, the differences were due to the route of administration, mainly intravenous infusion (see Table 3).

**Table 3:** Comparison of the medians of the different sections of the questionnaire according to profession or administration.

	p-value			
	**Preservation**	**Waste management**	**Transport**	**Administration**
Kruskal-Wallis test	0.151	0.127	0.134	0.015
Nurse-Pharmacist	0.224	0.165	0.693	0.067
Nurse-Occupational physician	0.224	0.342	0.168	0.022
Pharmacist-Occupational physician	0.564	0.384	0.168	0.623
	**Intravenous perfusion**	**Subcutaneous, intramuscular, intrathecal, intravenous bolus**	**Intravesical**	**Ophthalmic**
Kruskal-Wallis test	0.013	0.149	0.046	0.254
Nurse-Pharmacist	0.044	0.398	0.300	0.589
Nurse-Occupational physician	0.025	0.182	0.034	0.303
Pharmacist- Occupational physician	0.853	0.459	0.300	0.486

The statistics for the central measures, broken down by the different professions of the respondents, are provided in Table 4.

**Table 4:** Central statistics separated by section of the questionnaire and type of administration according to profession.

Item	Minimum	1st quartile	Median	Mean ± SD	3rd quartile	Maximum	Risk of exposure perception
**Nurse**							
Preservation	0	1	1	1.7 ± 1.2	2	4	Middle
Waste manag.	0	1	2	2.0 ± 1.2	3	4	Middle
Transport	0	1	1	1.5 ± 1.2	2	4	Middle
Administration	0	1	2	2.1 ± 1.2	3	4	High
Administration IV	0	1	2	2.1 ± 1.2	3	4	High
Administration SI	0	1	2	2.3 ± 1.2	3	4	High
Administration IVe	0	1.8	3	2.4 ± 1.2	3	4	High
Administration OP	0	1	2	2.1 ± 1.2	3	4	High
**Pharmacist**							
Preservation	0	1	1	1.3 ± 0.6	2	2	Middle
Waste manag.	0	1	2	1.6 ± 0.9	2	4	Middle
Transport	0	1	1	1.4 ± 0.9	2	4	Middle
Administration	0	1	2	1.9 ± 1.0	3	4	Middle
Administration IV	0	1	2	1.9 ± 1.0	3	4	Middle
Administration SI	0	1	2	2.0 ± 1.0	3	4	Middle
Administration IVe	0	1	2	2.2 ± 1.2	3	4	High
Administration OP	0	1	2	2.0 ± 1.0	3	4	Middle
**Occupational physician**							
Preservation	0	1	1	1.4 ± 1.1	1.8	2	Middle
Waste manag.	0	1	2	1.8 ± 1.0	2	4	Middle
Transport	0	1	1	1.1 ± 0.8	1	4	Middle
Administration	0	1	2	1.8 ± 1.0	2	4	Middle
Administration IV	0	1	2	1.8 ± 1.0	2	4	Middle
Administration SI	0	1	2	1.9 ± 1.0	2	4	Middle
Administration IVe	0	1	2	1.9 ± 1.0	2	4	Middle
Administration OP	0	1	2	1.8 ± 1.0	2	4	Middle
Global	0.05	1.1	1.9	2.0 ± 1.0	2.6	4	Middle

Waste manag. = Waste management

IV = Intravenous perfusion; SI = Subcutaneous, intramuscular, intrathecal, intravenous bolus: IVe = Intravesical; OP = Ophthalmic.

**Fig 2:**
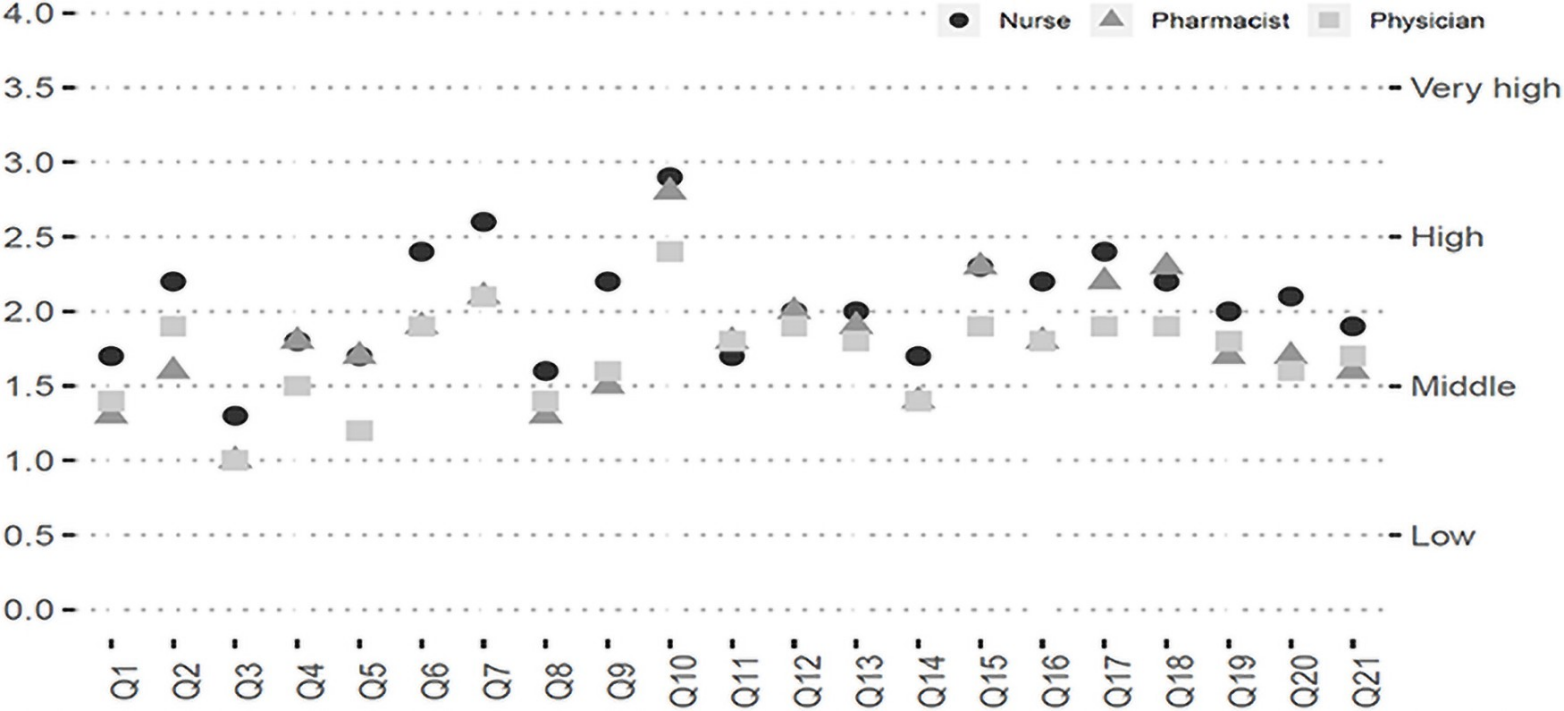
Average perception of risk of exposure values for the 21 items in the questionnaire, by profession.

The average perception of risk of exposure for each of the items in the questionnaire, disaggregated by profession, is provided in Fig 2.

## Discussion

Based on the responses obtained from health science professionals, this study was able to assess the perceived probability of exposure to HDs through their management by HHU and hospital units’ personnel. This study arises from the need to estimate the perception of the magnitude of the risk associated with HD handling, based on the current working conditions in most Spanish health centers, to determine, in later stages, the critical points of the process and establish adapted preventive measures that improve safety from the work point of view. To date and except for omission, no studies have been published in the literature that quantify this risk in the stages considered.

The good response rate achieved resulted from personalized recruitment, making a great effort to have representative participation of different professional groups in the health field with a high degree of knowledge about HDs (occupational physicians, specialized nurses in HD administration and hospital pharmacists). It is not surprising that the professional group with the most respondents was nursing staff because the stages considered in the questionnaire are fundamentally the responsibility of this group.

In addition, the high degree of knowledge of the participants served as validation for the results, and contributed to the low proportion of incomplete questionnaires. The questions that presented the greatest number of missing values (or blank responses) corresponded to items related to intravenous infusion administration, a route that has undergone a significant technological evolution in recent years and that has a wide range of devices and equipment; these devices and equipment have various levels of safety measures used in nonhomogeneous manners in different health centers around the country, which could lead to a lack of experience in the handling of any of these devices by the respondents.

Cronbach’s alpha (with values close to 1) confirmed the consistency of the questionnaire, which contributes robustness to the results. The ICC confirmed a high degree of agreement of the observations made by the different participants. These values guaranteed the consistency and reliability of the questionnaire used to determine the perception of risk of exposure to HDs.

### Results of the perception of risk of exposure to HDs

The results obtained, in the questionnaire as a whole as in most of the items separately, with scores that tended to the highest values, confirm the adequacy of the proposed probability scale compared to that of the United States pharmacopoeia, in the consensus statement on the management of HDs [10].

In this study, risk situations for workers that may arise during each stage of the process and with each type of device currently marketed have been explored. Thus, the item that presented the lowest risk of exposure was the one corresponding to transport of the HD to the place of administration (Q3), a logical result given that it was assumed that HDs are transported in airtight containers, minimizing the risk of damage to the HDs, on the one hand, and if damage does occur, containing spills or leaks that may occur from the primary container [19]. The item with the highest risk corresponded to the use of chambers without “*Air Stop*” filters during administration of HDs by intravenous infusion, a result that agrees with the opinion of other experts, who considered "flush operations" as high-risk actions [20]. These are special filters located in the drip chamber that prevent air from entering the infusion tube in the container containing the HD. Some conventional HD infusion systems are devoid of this type of filter, allowing the entry of air into the infusion line, forcing, if this occurs, the perfusion to stop and flushing of the trapped air, with the consequent risk of dripping or spilling the HD.

Other theoretically critical points in which there is a risk of accidental exposure during intravenous infusion administration are presented in the connection and disconnection of the infusion lines as well as in the area around the spike, where there is a risk of exposure by drops, or spills, by tears or breaks of the bag containing the HD or by an inadequate connection [20, 21]. Thus, the respondents rated as high risk the items corresponding to the connection of an HD to the infusion line (Q6) and the leakage of an HD through the spike that connects the extension to the HD container (Q7). However, low risk perception stands out, in the case of leakage in the puncture area, when the HD connection to the extension is made by *luer-lock* (Q8).

Regarding the disconnection points, the risk perception was lower than expected, with moderate risk, both in the tree-type infusion system (system with multiple bags or tubing connected through Y-site adapters) (Q12 and Q13) and in the valve infusion system (system that uses one single bag and tubing) (Q14). Notably, the perception of risk of exposure of the valve system *versus* the tree system was lower among the respondents, results that are in line with the preferences shown by nursing staff in previous studies [22], probably because they are simpler and more intuitive systems, as opposed to tree systems, which are complex to manage (numerous clamping and unclamping to administer an HD and wash sera) and have a high risk of accidental spills if personnel forget to clamp the secondary system. In addition, as a recent study showed, in some cases, safety measures can introduce new risks and dangers, not only for health personnel but also for the quality of the product and therefore for patient safety [23]. Thus, Costero et al. [24] demonstrated that in tree-type systems, when the drip chamber is empty, there is reflux of HD from the secondary system to the central serum washing system, creating danger in the disconnection process. Thus, the frequency with which the drip chamber is emptied and therefore a potential risk was moderate, according to the respondents (Q11).

Despite all of the above and although there is no solid evidence showing benefits of one system over the other, there is some consensus that the closed tree-type system presents fewer chances of exposure (as it guarantees the absence of disconnections during the administration process, and is therefore considered safer [20, 21].

The analysis of the results grouped by stages (preservation, transport, waste management and administration) showed that administration, followed by waste management, were the stages with the highest risk. This result is consistent with NIOSH considerations [3], which establish that the preparation of an HD (a stage not evaluated in this questionnaire because it is not performed in the home), together with administration and waste management, are the phases of the HD handling process with the highest occupational risk.

Considering the specific route of administration, intravesical instillation of HDs is the route that presents the greatest risk, followed by the administration of prefilled syringes (subcutaneous/intramuscular/intrathecal/intravenous bolus), both ahead of intravenous infusion or ophthalmic administration (administration of eye drops and intravitreal syringes). This result was very interesting because in recent years, the efforts of the industry to design and develop devices that help contain HD exposure have been mainly directed towards intravenous infusion administration (probably due to the inherent complexity of this type of administration and the greater frequency of use in the case of the HDs), leaving critical aspects of other administration routes of “minority” HDs less attended. Hence, it is logical and coherent that the perception of the risk of exposure of these routes is greater.

### Perception of risk of exposure according to professional group

Overall, the perception of the risk of exposure to HDs was higher for nursing staff, with significantly higher results when compared with both hospital pharmacists and the group of occupational physicians surveyed, finding no differences between the latter two. When these differences were analyzed according to the different stages of the process (sections of the questionnaire), it was observed that the weight of these fell in the administration phase, mainly in intravenous and intravesical perfusion administration, due to the discrepancies shown between occupational physicians and pharmacists compared to the nursing group.

Similarly, when each of the questions of the questionnaire was analyzed individually, the perception of risk of exposure by the nursing group was greater for all items except for 2, Q11 and Q18, the latter being related to the intravitreal administration of HDs, an activity carried out mainly by ophthalmologists. Although there was no participation in the questionnaire of ophthalmologists, both pharmacists and occupational physicians, based on their technical-theoretical knowledge, perceived these activities with a higher risk of exposure than the nursing group whose technical knowledge is based primarily on the practice of nursing care.

These results were not surprising because, as already mentioned above, most of the activities evaluated in the questionnaire are carried out, in clinical practice, by nursing staff; therefore, it is consistent that they present a greater perception of risk of exposure, based on the daily management of this type of medication and safety devices.

### Limitations

For the analysis of risk, a subjective estimate of the risk of exposure in different scenarios was made based on the perception of the participants in the questionnaire. Although perception may not reflect actual exposure, there are no incidental records or solid evidence that allows establishing the exact risk associated with each stage or procedure. Additionally, the quantitative assessment of HD exposure, considered the *gold standard* in the risk assessment of chemical agents [25], is not applicable to HDs, as it involves a validated sampling strategy and comparison with a reference limit value. In the case of HDs, there is no exposure threshold below which there is certainty that the harmful effect will not occur [26, 27].

## Conclusions

The perception of the risk of exposure associated with HD preservation, transport, administration, and waste management was generally moderate, thus tend to the mean values on the probability scale. This result was considered consistent because the assessment of the perception of risk of exposure was carried out considering the existing prevention measures (safety devices, closed system drug transfer devices and personal protective equipment). In turn, this fact demonstrated that the use of all these measures does not completely eliminate what workers consider as risky, and that there are still unresolved critical points in the process.

The perception of risk of exposure was higher for the nursing group for practically the entire questionnaire, the group responsible for most of the activities analyzed in the questionnaire and probably the group with the most knowledge of the real risks of the process and the critical potential exposure points therein. Nevertheless, the selection of different professional healthcare profiles in the survey allowed us to analyze the operations with the greatest difference in the risk perception between groups of professionals and thus enable us to establish preventive measures which better adapt to each situation.

For all the above, HDs should be integrated into a standardized management system, that is a comprehensive system for controlling the hazards associated with the HDs handling process, applicable to any hospital, that allows us to identify which points in the process have the greatest risk as well as how to control them by reporting each of the performed activities with the ultimate aim to improve the safety of patients and health professionals, with resource efficiency maximized and procedural incidents minimized, guaranteeing the quality and safety of the HD handling process in HHUs and hospital units.

It would be desirable that, in the future, a common methodology be followed for the risk assessment of HDs that allows not only monitoring effective preventive planning but also establishing a solid database for the evolution of working conditions that are produced so that preventive management and control can improve day by day. In this sense, having technologies applied to HDs that allow configuring specific management systems in relation to the management and traceability of HDs would provide enormous added value.

## Supporting information

S1 Table English version. (DOCX)

S2 Table Spanish version. (DOCX)
