# Supplementary material for: Perception of risk of exposure in the management of hazardous drugs in home hospitalization and hospital units
Source: PLoS One. 2021 Jul 1;16(7):e0253909. doi: 10.1371/journal.pone.0253909 (PMC8248625; doi:10.1371/journal.pone.0253909)
Supplement: S1 Table — (DOCX) [file pone.0253909.s001.docx]

| **S1 Table. Survey** |
| --- |
| **INTRODUCTION** |
| **Please, choose your profession in the drop-down menu:**   - Physician - Nurse - Pharmacist |
| **SPECIFIC QUESTIONS**  **(value from 0 to 4, with 0 being null probability and 5 being very high probability)** |
| 1. **CONSERVATION** |
| 1. During conservation, the probability of exposure to a hazardous drug (HD) due to loss of integrity of the container, producing a spill or splash, is considered to be: |
| 1. **WASTE MANAGEMENT** |
| 1. During waste management of an HD that did not met conservation conditions, the probability of exposure to the HD due to a loss of integrity of the container, producing a spill or splash, is considered to be: |
| 1. **TRANSPORT TO THE PLACE OF ADMINISTRATION** |
| 1. During transport to the place of administration, assuming that the HD is in an airtight container, the probability of exposure to the HD due to loss of integrity of the container, producing a spill or splash, is considered to be: |
| 1. **ADMINISTRATION: PREVIOUS COMMON STAGES** |
| 1. While maintaining the integrity of the container, prior to administration (checking that the container with the HD is airtight and does not leak), the probability of exposure to the HD in the case that the container was not adequate, is considered to be: |
| 1. During the return to the pharmacy service, in the case of HDs that do not present adequate integrity, the probability of exposure to the HD (assuming that it is transported in an airtight container) is considered to be: |
| 1. **ADMINISTRATION OF THE HAZARDOUS DRUG (HD) THROUGH INTRAVENOUS PERFUSION (IV)** |
| 1. In the case of using tree-type administration systems, during the connection of the HD to the main line of administration, indicate the probability of exposure due to a spill (because the extension to the HD is not adequately "clamped" or HD drips through the tip of the extension if it has not been flushed with diluent from the pharmacy service). |
| 1. When using tree-type administration systems, what is the probability of exposure to the HD due to a leak, made by the spike, in the HD connection to the extension (or secondary route)?    |
| 1. When using tree-type administration systems, what is the probability of exposure to the HD due to a leak in the "luer-lock" connection between the HD and extension (or secondary route)?   **** |
| 1. When using valve delivery systems, what is the probability of exposure to the HD due to a leak through the spike that connects the safety valve to the HD?   **** |
| 1. During the administration of the HD, when conventional drip chambers are used (without an "Air Stop" filter), there is a risk of air entering the administration line. As a consequence, it is necessary to stop the perfusion and flush the trapped air, with the consequent risk of dripping or spilling the HD through the tip of the main administration line that is connected to the patient. Indicate, therefore, the probability of exposure to the HD when these systems are used.    |
| 1. When an HD is administered, if the drip chamber remains empty, it is necessary to flush it manually to continue the perfusion. In this circumstance, the washing solution is contaminated by reflux of the HD. How often is the drip chamber empty? |
| 1. In the previous scenario, indicate the probability of dripping the contaminated wash solution when disconnecting the equipment from the patient, when the HD is administered with a pump. |
| 1. In the previous scenario, indicate the probability of dripping the contaminated wash solution when disconnecting the equipment from the patient, when the HD is administered by gravity. |
| 1. During the disconnection of the HD to the infusion line, when valve systems are used, the probability of exposure due to contamination with remnants of the HD in the safety valve is considered to be: |
| 1. **SUBCUTANEOUS, INTRAMUSCULAR, INTRATECAL OR INTRAVENOUS BOLUS ADMINISTRATION** |
| 1. Consider the existing probability of dripping at the tip of the syringe just before administration of the HD. |
| 1. During the administration itself, indicate the probability of exposure due to HD spillage or splashing and/or bag puncture. |
| 1. **INTRAVESICAL ADMINISTRATION** |
| 1. Once intravesical administration is complete, the administration equipment (syringe containing the HD + closed system, i.e., syringe + bladder probe) must be removed from the patient en bloc. Indicate the probability of exposure due to dripping of the HD through the probe tip. |
| 1. **OPHTHALMIC ADMINISTRATION** |
| 1. During ophthalmic administration, the probability of exposure due to spillage of the HD contained in the eye drops container is considered to be: |
| 1. During ophthalmic administration, the probability of exposure due to spillage of the HD contained in the intravitreal syringe is considered to be: |
| 1. **ADMINISTRATION: FINAL COMMON STAGES** |
| 1. Indicate the probability of contact with HDs that contaminate personal protective equipment (PPE). |
| 1. Indicate the probability of puncture when disposing of syringes with needles. |
