# Supplementary material for: Perception of risk of exposure in the management of hazardous drugs in home hospitalization and hospital units
Source: PLoS One. 2021 Jul 1;16(7):e0253909. doi: 10.1371/journal.pone.0253909 (PMC8248625; doi:10.1371/journal.pone.0253909)
Supplement: S2 Table — (DOCX) [file pone.0253909.s002.docx]

**S2 Table. Encuesta**

| **INTRODUCCIÓN** |
| --- |
| **Por favor, elija su profesión en el desplegable:**   - Médico / a - Enfermero /a - Farmacéutico / a |
| **PREGUNTAS ESPECÍFICAS**  **(valore de 0 a 4, siendo 0 *probabilidad nula* y 5 *probabilidad muy alta*)** |
| **CONSERVACIÓN** |
| 1. Durante la conservación, la probabilidad de exposición al MP debido a una pérdida de integridad del envase, que produzca un derrame o salpicadura, considera que es: |
| **GESTIÓN DE RESIDUOS** |
| 1. Durante la gestión de residuos de aquellos MP que no hayan cumplido las condiciones de conservación, la probabilidad de exposición al MP debido a una pérdida de integridad del envase, que produzca un derrame o salpicadura, considera que es: |
| **TRANSPORTE AL LUGAR DE LA ADMINISTRACIÓN** |
| 1. Durante el transporte al lugar de la administración, asumiendo que éste se realice dentro de un recipiente hermético, la probabilidad de exposición al MP debido a una pérdida de integridad del envase, que produzca un derrame o salpicadura, considera que es: |
| **ADMINISTRACIÓN: ETAPAS COMUNES PREVIAS** |
| 1. Durante el control de la integridad del envase, previo a la administración (comprobar que el envase que contiene el MP es hermético y no presenta fugas), la probabilidad de exposición al MP en el caso de que éste no fuese adecuado, considera que es: |
| 1. Durante la devolución al servicio de farmacia, para el caso de los MP que no presenten las condiciones adecuadas de integridad, la probabilidad de exposición al MP (asumiendo que se transporta en un recipiente o envase hermético) se considera que es: |
| **ADMINISTRACIÓN DEL MP MEDIANTE PERFUSIÓN IV** |
| 1. En el caso de utilizar sistemas de administración tipo árbol, durante la conexión del MP a la línea principal de administración, indique la probabilidad de exposición debido a un de derrame (debido a que la alargadera del MP no está adecuadamente clampada o existe goteo de MP a través de la punta de la alargadera si ésta no ha sido purgada con diluyente desde servicio de farmacia) |
| 1. Cuando utiliza sistemas de administración tipo árbol, cuál la probabilidad de exposición al MP, debido a una fuga a través de la conexión del MP a la alargadera (o vía secundaria) en el caso de que ésta se realice mediante punzón    |
| 1. Cuando utiliza sistemas de administración tipo árbol, cuál la probabilidad de exposición al MP, debido a una fuga a través de la conexión del MP a la alargadera (o vía secundaria) en el caso de que ésta se realice mediante conexión “luer-lock”:   **** |
| 1. Cuando utiliza sistemas de administración valvulares, cuál es la probabilidad de exposición al MP, debido a una fuga a través del punzón que conecta la válvula de seguridad del sistema valvular al MP:   **** |
| 1. Durante la administración del MP, cuando se utilizan cámaras de goteo convencionales (sin filtro "*Air Stop*"), existe riesgo de entrada de aire en la línea de administración. Como consecuencia, es necesario parar la perfusión y purgar el aire atrapado en ésta, con el consiguiente riesgo de goteo o derrame del MP a través de la punta de la línea principal de administración que se conecta al paciente. Indique por tanto, la probabilidad de exposición al MP cuando se utilizan estos sistemas.    |
| 1. Cuando se administra un MP, si la cámara de goteo se queda vacía, es necesario purgarla manualmente para continuar con la perfusión. En esta circunstancia, la solución de lavado se contamina por reflujo del MP, ¿con que frecuencia se queda la cámara de goteo vacía? |
| 1. Situándose en el escenario anterior, indique la probabilidad de goteo de la solución de lavado contaminada al desconectar el equipo del paciente, cuando el MP se administra con bomba. |
| 1. Situándose en el escenario anterior, indique la probabilidad de goteo de la solución de lavado contaminada al desconectar el equipo del paciente, cuando el MP se administra por gravedad |
| 1. Durante la desconexión del MP a la línea de infusión, cuando se utilizan sistemas valvulares, la probabilidad de exposición por contaminación con restos del MP de la válvula de seguridad del sistema valvular, considera que es: |
| **ADMINISTRACIÓN SUBCUTÁNEA, INTRAMUSCULAR, INTRATECAL O BOLO INTRAVENOSO** |
| 1. ¿Qué probabilidad existe de que exista goteo en la punta de la jeringa justo antes de la administración del MP? |
| 1. Durante la propia administración, indique la probabilidad de exposición debido a derrame del MP, salpicadura y/o pinchazo. |
| **ADMINISTRACIÓN INTRAVESICAL** |
| 1. Una vez finalizada la administración intravesical, el equipo de administración (jeringa que contenía el MP + sistema cerrado de conexión a la jeringa + sonda vesical) se ha de retirar del paciente en bloque. Indique la probabilidad de exposición debido a goteo del MP a través de la punta de la sonda. |
| **ADMINISTRACIÓN OFTÁLMICA** |
| 1. Durante la administración oftálmica, la probabilidad de exposición debido a derrame del MP contenido en el envase de colirio, considera que es: |
| 1. Durante la administración oftálmica, la probabilidad de exposición debido a derrame del MP contenido en la jeringa intravítrea, considera que es: |
| **ADMINISTRACIÓN: ETAPAS COMUNES FINALES** |
| 1. Indique la probabilidad de contacto con restos del MP que hayan quedado contaminando los equipos de protección individual (EPI). |
| 1. Indique la probabilidad de pincharse al eliminar en contenedores las jeringas con aguja: |
| Anexo 1. Encuesta |
